# Determinants of food safety knowledge and practices among food handlers in Bangladesh: An institution-based cross-sectional study

**DOI:** 10.1016/j.heliyon.2024.e25970

**Published:** 2024-02-10

**Authors:** Aysha Siddiky, Kakali Mollick, Md. Aktarujjaman, Farhadul Islam, Mohammed A. Mamun, Nitai Roy

**Affiliations:** a Faculty of Nutrition and Food Science, Patuakhali Science and Technology University, Patuakhali, 8602, Bangladesh; b Dept. of Biochemistry and Molecular Biology, University of Rajshahi, Bangladesh; c CHINTA Research Bangladesh, Dhaka, Bangladesh; d Department of Public Health and Informatics, Jahangirnagar University, Dhaka, Bangladesh; e Department of Biochemistry and Food Analysis, Patuakhali Science and Technology University, Patuakhali, 8602, Bangladesh

**Keywords:** Food handlers, Food safety, Hygienic practices, Bangladesh

## Abstract

The engagement of a large number of people in big-scale cooking raises the danger of food contamination due to incorrect handling, whether deliberate or unintentional. Contamination during large-scale production poses a serious hazard to consumer health and has significant financial implications for a nation. This study aimed to investigate the food safety knowledge and practices of institutional food handlers in Bangladesh, considering the growing concern surrounding this issue and the lack of available information on foodborne illnesses related to institutions. In addition, the study aimed to determine the factors influencing both knowledge and practices. A cross-sectional study was conducted from June to September 2022, involving 408 institutional food handlers. The sample size was determined using Cochran's formula, and data was collected through purposive sampling. The participants were interviewed in person and completed a pilot-tested questionnaire. A multiple linear regression analysis was conducted to determine the factors related to food safety knowledge and practices. The majority of participants were female (71.3%) and aged between 26 and 35 (mean age 34.53 ± 9.06 years). They were most knowledgeable about hand hygiene and food separation but lacked knowledge about foodborne pathogens and food storage. Thawing food at room temperature was the most inappropriate practice (86%). The mean scores for knowledge and practice were found to be 16.11 ± 2.76 on a 26-point scale (61%), and 9.59 ± 2.07 on a 15-point scale (64%), respectively. Rural food handlers, those with higher education, working more than 10 h per day, and being familiar with HACCP, had higher knowledge. Food handlers aged 18 to 25, with higher income, working in private institutions, having food safety authority knowledge, actively engaging in food safety training, working more than 10 h per day, and having a positive health perception, had better food safety practices.The results of this study reinforce the notion that institutional food handlers would benefit from enhanced exposure to food safety interventions, active participation in training sessions, and strict adherence to food hygiene regulations in their food handling knowledge and practices.

## 1 Introduction

Institutions, including educational establishments such as schools, colleges, universities, and medical facilities like hospitals, prisons, and military bases, play a vital role in the food service sector by providing food services [1,2]. This catering sector has the potential to offer essential nourishment to a significant portion of the population, especially children and the elderly. However, the increasing scale and complexity of these operations raise concerns about hygiene lapses, which can have severe public health consequences if strict hygiene standards are not maintained [[3], [4], [5]]. The global food service sector has seen substantial growth due to changing lifestyles and increased incomes [6]. However, both commercial and non-commercial foodservice outlets, including universities and hospitals, face challenges in ensuring food safety. Foodborne illnesses in these settings result from factors like poor cooking practices, extended food preparation-to-consumption times, inadequate heat treatment, infectious personnel, contaminated materials, subpar equipment cleaning, cross-contamination, and low-quality ingredients, including food residue [7].

Numerous studies conducted worldwide have explored the knowledge, attitudes, and practices of institutional food handlers in various countries; for instance, Ghana [3], Kenya [8], Pakistan [7], Lebanon [9], Saudi Arabia [10], Malaysia [11], Ethiopia [12], and India [13]. Some studies have identified noteworthy connections between knowledge of food safety and various sociodemographic factors including age, food safety training, level of education, work experience, and the type of institution [9,10,13]. Additionally, there has been strong evidence linking food safety practices to educational attainment, training in food safety, work experience, and the type of institution [9,10,13]. In Bangladesh, a number of studies have assessed the knowledge, attitudes, and practices (KAP) regarding food safety among different groups, including students [[14], [15], [16]], household food handlers [17,18], workers of baking industries [19], fish farmers and restaurant food handlers [20], and chicken vendors [21]. However, there is a scarcity of studies investigating the KAP of institutional food handlers. For instance, Al Banna et al. (2022) specifically delved into the food safety KAP of hospital service staff in Bangladesh [22], which warrants a knowledge gap to the sector.

Previous studies link foodborne outbreaks in universities to factors like suboptimal food handling, inadequate heat treatment, and low-quality ingredients [23,24]. In Bangladesh, both public and private university students rely on daily meals, but food quality has declined significantly, and there is a risk of contamination [25]. Unfortunately, university food establishments often lack inspections, increasing the risk of foodborne infections. Food safety is becoming an increasingly important issue in military operations [26]. In the German military, for instance, a retrospective long-term analysis of food-borne outbreaks conducted between 1995 and 2019 revealed that 235 different items were contaminated with food-borne microorganisms. The most common harmful bacteria found in food samples were *Salmonella* spp. (19.2%) and *Bacillus cereus* (42.2%) [27]. The community kitchen practice prevalent in the military poses a high risk of food contamination [28]. In Bangladesh, a study observed varying food safety practices among military food handlers [29], but it had limitations in sample size and coverage of food safety knowledge. Furthermore, patients with weakened immunity in healthcare facilities face higher foodborne infection risks, but their food safety often receives less attention. Overcrowding and limited facilities like handwashing stations in Bangladeshi healthcare settings add to the challenges [30]. A study at a cancer research facility found low patient satisfaction with meals, indicating the need for improvement [31]. Inadequate monitoring and funding in healthcare facilities contribute to illnesses among hospital workers and caregivers [30].

The assessment of the knowledge and practices exhibited by individuals responsible for handling food in institutional settings plays a pivotal role in the advancement and enforcement of food safety protocols within hospitals, university cafeterias, and military facilities situated in Bangladesh. Hence, the aim of this study is to evaluate the level of knowledge and adherence to food safety practices among individuals responsible for handling food within institutional settings. Additionally, we hypothesize that differences in food safety knowledge and practices are likely to exist based on demographics and work-related factors such as age, education level, work experience, institution type, food safety training, and familiarity with the HACCP system. Our study aims to answer these questions by conducting a thorough evaluation across various institutions, carefully examining the factors that have a significant impact, and providing valuable insights to bridge a specific knowledge gap in the current literature on food safety in Bangladesh. This study will also offer a valuable contribution to the academic discussion and has practical consequences for policymakers, academics, and practitioners in the areas of public health and food safety.

## 2 Methods

### 2.1 Study setting

This study was conducted among food handlers from one of the largest cantonments, dining halls, and cafeterias of six public universities, two public hospitals, and two private hospitals (a total of eleven institutions from the public and private sectors) located in the districts of Dhaka (capital), Barishal, Khulna, Jessore, Patuakhali, Sirajganj, and Manikganj districts of Bangladesh. In various institutions, including universities, hospitals, and military facilities, there are typically unique work methodologies, organizational structures, and food-handling protocols in place. To encompass a wide variety of practices and challenges faced by food handlers in different work environments, we made sure to include a range of institutions. We also include a variety of institutions from various districts in Bangladesh to ensure that our findings are not limited to a certain region or type of institution, increasing the external validity and generalizability of our findings.

### 2.2 Study design, participants, and sampling

A cross-sectional study was carried out in selected institutions in Bangladesh from June to September 2022. According to the Codex Alimentarius definition, a food handler is an individual who is responsible for directly handling both packaged and unpackaged food, as well as food equipment, utensils, and food contact surfaces, and is therefore accountable for ensuring that these items are clean and safe for human consumption [32]. Therefore, in this assessment, we defined food handlers as those who directly handle raw fish, meat, chicken, and vegetables, who work to cook food in the kitchen, dish cleaners, table and food storage cleaners, and food caterers, or waiters. Food handlers must be at least 18 years old and of Bangladeshi by birth to be eligible for the study. The research excluded any food handlers who were absent due to illness during the survey period. This includes food handlers who are on sick leave or who have been diagnosed with an infectious disease that could pose a risk to the study. The participants in this study were chosen using a purposive sampling strategy.

### 2.3 Sample size calculation

The formula for a cross-sectional study, n = Z^2^PQ/D^2^, was used to determine the sample size for this investigation. In this equation, n stands for the necessary sample size, Z for a 95% confidence interval, D for the 5% (SD = 0.05) margin of error, and Q for 1-P. Due to the absence of a comparative study among institutional food handlers, the value of P was assumed to be 50%. The minimum sample size is therefore 384. To enhance the reliability and applicability of this study, the researchers included more participants.

### 2.4 Interviews and data collection

Before collecting data, a questionnaire and a letter elucidating the study's objectives, significance, and inquiry method were sent to the hall administrations of each university, the management of the hospital, and the administration of the cantonment. An interview-based survey was carried out after receiving approval from the relevant facilities. The head of the kitchen was approached in person or by phone to arrange the best time for data collection. Data were collected by trained interviewers who visited the sites. The study's principal investigator organized an offline training session to brief the data collectors on the survey's various components, as well as on interview methodologies and inclusion/exclusion criteria. The interview was conducted at a time convenient for food handlers so that their work would not be affected. Preceding the commencement of data collection, the questionnaire underwent a rigorous process of independent translation from English to Bangla, carried out by a proficient bilingual expert. Following that, the questionnaire in Bangla was translated back into English by another expert who is fluent in both languages. There were no notable differences in meaning detected between the original and back-translated iterations. To evaluate the questionnaire's reliability, a pilot study was undertaken, encompassing a limited sample size of 20 food handlers. Following the analysis of the reliability test outcomes, adjustments were implemented to the questionnaire to optimize its reliability and validity. Food handlers were required to provide their informed consent by signing a consent form, or permission was obtained verbally from persons who couldn't read or write. Food handlers share sociodemographic information, knowledge, and practices about food safety and information about jobs and training. Each interview took about 15–20 min, and each respondent was assigned a unique code to ensure confidentiality. The Cronbach's alpha value calculated for this study was 0.71, which was satisfactory for questionnaire validity [33]. Based on the cut-off outlined by Bloom, the levels of knowledge and practices exhibited by the study participants were classified into three distinct categories: high (80.0%–100.0%), moderate (60.0–79.9%), and low (less than 60.0%) [34].

### 2.5 Study variables and measures

The content and structure of the questionnaire were adapted based on prior research studies [3,22,35]. The questionnaire consisted of four distinct sections, encompassing a total of 61 closed-ended questions.

The first section (A) focuses on sociodemographic factors, occupational characteristics, and training-related elements, including gender, age, religion, marital status, income, educational background, institution type (derived from a sample of eleven institutions), employment experience, and working hours. It also assesses respondents' knowledge of the Hazard Analysis and Critical Control Points (HACCP) system, food safety authorities, participation in food safety training programs, and overall health status. The second section (B) assesses job satisfaction among employees, while the third section (C) investigates respondents' food safety knowledge. The final section (D) focuses on respondents' food safety practices.

#### 2.5.1 Food safety knowledge

The food safety knowledge section consisted of twenty-six closed-ended questions that assessed participants' knowledge of personal hygiene, cross-contamination, food-borne illnesses, food-borne microorganisms, temperature control, and hygiene practices. Every question offered "true," "false," or "don't know," response alternatives. Except for questions 10 and 14, "true" responses received 1 point, while "false" and "don't know" responses received 0 points. For queries 10 and 14, "False" received 1 point, while "True" and "Don't know" received 0 points. Each participant's total scores in this section ranged from 0 to 26, with higher scores indicating greater food safety knowledge.

#### 2.5.2 Food safety practices

The food safety practice section emphasized the significance of personal hygiene, hand-washing, and preventing infectious diseases and cross-contamination. This section included 15 ″yes" or "no." questions. Each correct practice received 1 point, while each incorrect practice received 0. For questions 7, 11, 12, 13, and 14, "yes" was awarded 0 points and "no" 1 point. This section assigned each participant a score from 0 to 15, with higher scores indicating more food safety adherence to food safety practices.

### 2.6 Statistical analysis

Data analysis in this study was carried out utilizing SPSS (IBM version 27.1.0, New York, USA). The analysis encompassed the computation of descriptive statistics, including percentages, mean values, and standard deviations. A normality test was executed for each variable to evaluate the distribution of dependent variables. Given the skewed distribution of the data, the comparison of means was conducted using the Kruskal-Wallis H test and the Mann-Whitney *U* test. The Kruskal-Wallis H test was utilized to compare three or more independent groups, whilst the Mann-Whitney *U* test was employed to compare two independent groups. To assess multicollinearity, GVIF and tolerance were examined, with a threshold value of 10 [36]. In this study, multiple linear regression models were employed to explore the relationships between Knowledge and practice and various sociodemographic, occupational, and educational variables. After fitting the models, a thorough assessment of all assumptions related to linear regression was conducted. The strength of associations was quantified using regression coefficients (β), accompanied by 95% confidence intervals. Statistical significance was established with a p-value less than 0.05.

## 3 Results

### 3.1 Demographic and employment-related characteristics

The characteristics and employment details of the participants in this research are summarized in Table 1. Out of the 408 food handlers included in the study, 71.3% were female, and 41.7% fell within the age range of 26–35 (mean age 34.53 ± 9.06 SD). A significant majority, 72.8%, resided in urban areas, while 47.1% reported a monthly income between 15,000 and 30,000 Bangladeshi taka (equivalent to 140.74 to 281.48 USD). In terms of educational attainment, 39.7% had a secondary-level education. Regarding employment, 90.2% were associated with public organizations, and 26.5% had less than 5 years of professional experience in the food service industry. A substantial portion, 56.6%, worked full-time, and 72.1% dedicated over 10 h to work each day. Notably, 65.2% had no awareness of food safety authority, and approximately 90.4% of participants had not received any food safety training.

**Table 1:** Sociodemographic and employment-related characteristics.

Characteristics	Frequency	Percent (%)
Gender		
Female	291	71.3
Male	117	28.7
**Age in year**		
18 to 25	70	17.2
26 to 35	170	41.7
36 to 45	117	28.7
Above 46	51	12.5
**Residence**		
Urban	297	72.8
Rural	111	27.2
**Religion**		
Islam	347	85
Hinduism	61	15
**Marital status**		
Unmarried	87	21.3
Married	321	78.7
**Monthly income (in BDT)**		
Below 15000	182	44.6
15000 to 30000	192	47.1
Above 30000	34	8.3
**Education**		
No formal education	17	4.2
Primary	156	38.2
Secondary	162	39.7
Higher secondary or above	73	17.9
**Institution type**		
Public	368	90.2
Private	40	9.8
**Employment experience (Years)**		
Below 5	108	26.5
5 to 10	155	38
Above 10	145	35.5
**Employment type**		
Part-time	7	1.7
Full time	231	56.6
Temporal	170	41.7
**Working hours per day**		
Up to 10	114	27.9
Above 10	294	72.1
**Idea about the HACCP system**		
No	370	90.7
Yes	38	9.3
**Idea about food safety authority**		
No	266	65.2
Yes	142	34.8
**Food safety training**		
No	369	90.4
Yes	39	9.6
**Health perception**		
Poor	90	22.1
Average	214	52.5
Good	104	25.5

### 3.2 Work satisfaction of food handlers

Workplace satisfaction was assessed to see how happy institutional food handlers were with their professional environment, coworkers, and how others perceived them (Table 2). Interestingly, 35.8% of institutional food handlers said they would not choose the same job again, while 23.5% said they had no opinion. However, 40.7% of respondents said they would return to the same profession if given the chance. Approximately 90% of respondents said they would accept it if another job offered them something better. Half of the respondents (51.2%) said their workplace met all of the requirements for food safety, while 26.7% said it did not. Almost 84.6% of food handlers stated they were respected by their coworkers. Similarly, 85.5% of food handlers stated that the supplied meal posed no health danger to the people, while 9.8% were unaware of the issue.

**Table 2:** Work satisfaction of institutional food handlers in Bangladesh.

Statements	Responses, n (%)		
	No	Yes	Do not know
If given the opportunity to select a profession, would you want to pursue the same profession?	146 (35.8)	166 (40.7)	96 (23.5)
If presented with a more advantageous employment opportunity, would you be inclined to accept it?	32 (7.8)	367 (90)	9 (2.2)
Does the workplace possess all of the necessary conditions to ensure the upkeep of food safety standards?	109 (26.7)	209 (51.2)	90 (22.1)
Are the other employees of the institution respectful of the kitchen staff?	26 (6.4)	345 (84.6)	37 (9.1)
Are the foods served to individuals hazardous to their health?	349 (85.5)	19 (4.7)	40 (9.8)

### 3.3 Food safety knowledge and its associated factors among institutional food handlers in Bangladesh

Table 3 provides an overview of the knowledge levels among food handlers. The results reveal that most food handlers have a strong understanding of the importance of hygiene practices, including handwashing after various activities and before meal preparation. Approximately 96.3% of food handlers identify handwashing as an effective measure to reduce food contamination risk. 83.8% of food handlers are aware of wearing gloves while handling food to minimize contamination risks. In terms of cross-contamination knowledge, 94.9% of the food handlers accurately stated that it is crucial to keep raw and cooked foods separate to prevent cross-contamination, with 76.5% aware of contaminated food causing bloody diarrhea and 48.5% aware of typhoid fever. However, most food handlers are unclear about foodborne pathogens like Salmonella, E. coli, Shigella, Bacillus cereus, and Hepatitis A. Only 39.7% of food handlers have relatively lower familiarity with food storage temperature and its impact on food safety.

**Table 3:** Food safety knowledge of institutional food handlers in Bangladesh.

Statements	Responses, n (%)		
	True	False	Don't know
1) It is critical to wash your hands after dealing with currency.	**408 (100)**	0 (0)	0 (0)
2) It is essential to cleanse your hands upon wiping down a table.	**408 (100)**	0 (0)	0 (0)
3) It is imperative to cleanse your hands after sneezing.	**406 (99.5)**	0 (0)	2 (0.5)
4) It is essential to cleanse your hands after using the toilet.	**408 (100)**	0 (0)	0 (0)
5) The time required to wash your hands is about 20 s.	**347 (85)**	29 (7.1)	32 (7.8)
6) Prior to making a meal, washing your hands is crucial.	**405 (99.3)**	0 (0)	3 (0.7)
7) It's crucial to wash your hands following the handling of raw meat.	**407 (99.8)**	0 (0)	1 (0.2)
8) Handwashing minimises the chance of food contamination both before and after handling raw food.	**393 (96.3)**	3 (0.7)	12 (2.9)
9) Wearing gloves while handling food helps to reduce the chance of food contamination.	**342 (83.8)**	13 (3.2)	53 (13)
10) Utensils that have been cleaned with detergent increase the risk of contamination.	151 (37)	**172 (42.2)**	85 (20.8)
11) Eating and drinking during raw food handling increases the risk of food contamination.	**257 (63)**	33 (8.1)	118 (28.9)
12) Raw and cooked foods should be kept separate to minimize cross-contamination.	**387 (94.9)**	3 (0.7)	18 (4.4)
13) Typhoid fever can be passed from person to person through the consumption of contaminated food.	**198 (48.5)**	51 (12.5)	159 (39)
14) AIDS is transmissible through contaminated food.	135 (33.1)	**97 (23.8)**	176 (43.1)
15) Bloody diarrhea is transmissible through contaminated food.	**312 (76.5)**	21 (5.1)	75 (18.4)
16) Salmonella is regarded as one of the various food-borne pathogens.	**27 (6.6)**	3 (0.7)	378 (92.6)
17) E. coli is regarded as one of the various food-borne pathogens.	**20 (4.9)**	3 (0.7)	385 (94.4)
18) Shigella is regarded as one of the various foodborne pathogens.	**15 (3.7)**	5 (1.2)	388 (95.1)
19) Bacillus cereus is regarded as one of the various foodborne pathogens.	**15 (3.7)**	4 (1)	389 (95.3)
20) Hepatitis A is regarded as one of the various foodborne pathogens.	**78 (19.1)**	29 (7.1)	301 (73.8)
21) Microbes can be found on the skin, nose, and mouth of even the healthiest food handlers.	**277 (67.9)**	62 (15.2)	69 (16.9)
22) The ideal temperature for storing perishable food is 5 °C.	**244 (59.8)**	23 (5.6)	141 (34.6)
23) The storage temperature for hot, ready-to-eat food is 65 °C.	**162 (39.7)**	31 (7.6)	215 (52.7)
24) The freezing process effectively inactivates all bacteria that have the potential to cause foodborne illnesses.	**132 (32.4)**	43 (10.5)	233 (57.1)
25) Raw meat should be stored on the bottom shelf of the refrigerator.	**257 (63)**	36 (8.8)	115 (28.2)
26) Storing raw and cooked food together causes poisoning.	**397 (97.3)**	0 (0)	11 (2.7)

Note: Bold indicates a correct answer.

The overall accuracy rate on the knowledge exam was 61% (16.11 out of 26, multiplied by 100), indicating a moderate level of knowledge. The mean score was 16.11 on a scale of 26.0 (SD = 2.76, range: 9–26). Noteworthy variations in the mean score for food safety knowledge were observed across various sociodemographic and employment-related factors, such as place of residence, religion, level of education, type of institution, number of daily working hours, familiarity with the HACCP system, and self-perceived health (Table 5).

The findings from the linear regression analysis indicate that there is a statistically significant difference in food safety knowledge ratings between food handlers living in urban regions and those living in rural areas (β = −0.22; p = 0.001). Individuals with lower levels of education, including those who have not received any formal education (β = −0.18, p = 0.001), individuals with primary education (β = −0.36, p = 0.000), and individuals with secondary education (β = −0.23, p = 0.001), demonstrated a diminished comprehension of food safety in comparison to those with a higher level of education (higher secondary or above). Food handlers working in institutional settings for more than 10 h per day demonstrated higher knowledge scores compared to those working fewer than 10 h per day (β = 0.14, *p* = 0.008). Additionally, participants who had some familiarity with the HACCP system exhibited higher levels of knowledge (β = 0.16, *p* = 0.003) than those who did not.

### 3.4 Food safety practices and their associated factors among institutional food handlers in Bangladesh

Table 4 provides a summary of the practices followed by food handlers. It shows that 97.8% of food handlers clean their hands before and after handling fresh and unwrapped food items, while 96.6% wash their hands before and after dealing with cooked unwrapped food. After chopping fresh chicken or meat, 98.5% wash and disinfect the knife. The majority of respondents (99.3%) sanitize food-contact surfaces before and after food preparation. However, 86% defrost food at room temperature, 76% don't wear gloves, 77% don't wear an apron, and 41.9% don't stay home when sick.

**Table 4:** Food safety practices of institutional food handlers in Bangladesh.

Statements	Responses, n (%)	
	Yes	No
1) Do you always wash your hands before and after working with unpackaged, raw foods?	**399 (97.8)**	9 (2.2)
2) Do you always wash your hands both prior to and subsequent to handling unpackaged prepared food?	**394 (96.6)**	14 (3.4)
3) Do you wear gloves while preparing food?	**98 (24)**	310 (76)
4) When touching or passing out unwrapped foods, do you wear an apron?	**94 (23)**	314 (77)
5) When touching or passing out unwrapped food, do you use a mask?	**135 (33.1)**	273 (66.9)
6) Do you cover your hair when touching or distributing unwrapped food?	**155 (38)**	253 (62)
7) Do you let your fingernails grow?	23 (5.6)	**385 (94.4)**
8) Do you rinse vegetables prior to slicing them?	**255 (62.5)**	153 (37.5)
9) Do you utilize separate cutting boards for fresh vegetables fruits and raw meat?	**314 (77)**	94 (23)
10) Do you clean and disinfect the knife after slicing raw meat or chicken?	**402 (98.5)**	6 (1.5)
11) Do you work when you are suffering from infectious diseases (flu, cold, diarrhea, coughing, etc.)?	171 (41.9)	**237 (58.1)**
12) If you have lesions or cuts on your hands, do you continue work?	130 (31.9)	**278 (68.1)**
13) Do you allow food to thaw at room temperature?	351 (86)	**57 (14)**
14) Do you leave prepared food out at room temperature for longer than 4 h?	105 (25.7)	**303 (74.3)**
15) Do you wash food contact surfaces prior to and following food preparation?	**405 (99.3)**	3 (0.7)

Note: Bold indicates a correct answer.

**Table 5:** Mean score food safety knowledge and practices by demographic and employment-related characteristics.

Characteristics	Food safety knowledge	*p-value*^*†*^	Food safety practice	*p-value*^*†*^
Gender				
Female	16.10 ± 2.78	0.830	9.87 ± 2.11	0.000
Male	16.13 ± 2.72		8.89 ± 1.79	
**Age in year**				
18 to 25	16.47 ± 2.49	0.201	10.00 ± 2.15	0.026
26 to 35	16.34 ± 3.04		9.48 ± 2.09	
36 to 45	15.76 ± 2.61		9.75 ± 1.93	
Above 46	15.61 ± 2.36		8.98 ± 2.10	
**Residence**				
Urban	15.88 ± 2.80	0.002	9.56 ± 2.15	0.477
Rural	16.72 ± 2.57		9.67 ± 1.86	
**Religion**				
Islam	16.00 ± 2.81	0.031	9.60 ± 2.04	0.870
Hinduism	16.70 ± 2.37		9.51 ± 2.24	
**Marital status**				
Unmarried	16.56 ± 2.91	0.169	10.07 ± 2.12	0.014
Married	15.98 ± 2.71		9.45 ± 2.04	
**Monthly income (in BDT)**				
Below 15000	15.79 ± 2.72	0.129	8.86 ± 1.91	0.000
15000 to 30000	16.44 ± 2.72		9.95 ± 2.05	
Above 30000	15.94 ± 3.05		11.44 ± 1.13	
**Education**				
No formal education	15.47 ± 2.35	0.000	9.53 ± 1.84	0.000
Primary	15.40 ± 2.58		8.71 ± 1.67	
Secondary	16.22 ± 2.67		9.83 ± 2.14	
Higher secondary or above	17.49 ± 2.91		10.95 ± 1.87	
**Institution type**				
Public	15.95 ± 2.76	0.000	9.41 ± 2.05	0.000
Private	17.58 ± 2.35		11.20 ± 1.44	
**Employment experience (Years)**				
Below 5	16.32 ± 2.46	0.058	9.59 ± 2.17	0.663
5 to 10	16.38 ± 2.94		9.45 ± 1.85	
Above 10	15.65 ± 2.73		9.73 ± 2.22	
**Employment type**				
Part-time	16.43 ± 4.04	0.076	10.29 ± 1.60	0.000
Full time	16.37 ± 2.79		10.21 ± 2.06	
Temporal	15.73 ± 2.63		8.71 ± 1.78	
**Working hours per day**				
Up to 10	15.39 ± 2.52	0.001	8.68 ± 1.72	0.000
Above 10	16.38 ± 2.80		9.94 ± 2.09	
**Idea about the HACCP system**				
No	15.94 ± 2.73	0.000	9.35 ± 1.98	0.000
Yes	17.74 ± 2.59		11.89 ± 1.48	
**Idea about food safety authority**				
No	15.89 ± 2.68	0.066	9.18 ± 2.02	0.000
Yes	16.50 ± 2.88		10.35 ± 1.94	
**Food safety training**				
No	16.04 ± 2.75	0.128	9.38 ± 1.99	0.000
Yes	16.69 ± 2.78		11.51 ± 1.79	
**Health perception**				
Poor	15.49 ± 2.64	0.026	8.71 ± 1.68	0.000
Average	16.11 ± 2.58		9.30 ± 1.99	
Good	16.63 ± 3.11		10.93 ± 1.91	
**Total**	16.11 ± 2.76		9.59 ± 2.07	

**Note:**^†^The Kruskal-Wallis H and Mann-Whitney U tests were used to compute p-values. The bolded values indicate the level of statistical significance (*p* < 0.05).

The practice test revealed a 64% (9.59 out of 15, multiplied by 100) correct response rate, indicating moderate proficiency in food safety practices among participants. The mean score for food safety practices was 9.59 on a 15-point scale (SD = 2.07, range: 5–14). All other factors, except the place of residence, religion, and employment experience, significantly influenced the mean score of food safety practices (all *p* < 0.05) (Table 5).

The results of the study indicate that food handlers between the ages of 18 and 25 had notably higher scores in food safety practices when compared to food handlers aged 46 years and older (β = 0.16, *p* = 0.040). Food handlers with a monthly income below 15,000 TK (β = −0.34, *p* = 0.001) and between 15,000 and 30,000 TK (β = −0.16, *p* = 0.050) exhibited significantly lower practice scores than those with incomes exceeding 30,000 TK. Participants working in government institutions displayed lower food safety practice scores compared to those employed in private institutions (β = −0.31, *p* = 0.000). Food handlers working more than 10 h per day in an institution demonstrated better food safety practices than those working up to 10 h per day (β = 0.1, *p* = 0.017). Knowledge of the HACCP system (β = 0.13, *p* = 0.003), awareness of the food safety authority (β = 0.11, *p* = 0.006), and receiving food safety training (β = 0.09, *p* = 0.032) were significantly associated with higher food safety practice scores. In addition, participants with a positive perception of their health demonstrated more effective food safety practices (β = 0.17, p = 0.004) than those with a negative perception of their health. Participants' food safety knowledge was significantly related to their food safety practices (*p* = 0.006) (Table 6).

**Table 6:** Determinants of food safety knowledge and practices.

Variables	Food safety knowledge†				Food safety practice‡			
	Unstandardized (B)	β	*p-value*	95% CI	Unstandardized (B)	β	*p-value*	95% CI
Age in year								
18 to 25	−0.25	−0.03	0.718	−1.6, 1.1	0.86	0.16	0.040	0.04, 1.68
26 to 35	−0.34	−0.06	0.511	−1.34, 0.67	0.44	0.11	0.156	−0.17, 1.05
36 to 45	−0.56	−0.09	0.224	−1.46, 0.34	0.5	0.11	0.075	−0.05, 1.05
Above 46	RC				RC			
**Gender**								
Female	−0.52	−0.09	0.111	−1.15, 0.12	0.2	0.04	0.315	−0.19, 0.59
Male	RC				RC			
**Residence**								
Urban	−1.35	−0.22	0.001	−2.13, −0.57	0.16	0.03	0.528	−0.33, 0.64
Rural	RC				RC			
**Monthly income (in BDT)**								
Below 15000	0.62	0.11	0.374	−0.75, 1.98	−1.42	−0.34	0.001	−2.25, −0.59
15000 to 30000	0.96	0.18	0.084	−0.13, 2.06	−0.67	−0.16	0.050	−1.33, 0
Above 30000	RC				RC			
**Religion**								
Islam	−0.72	−0.09	0.054	−1.45, 0.01	0.04	0.01	0.876	−0.41, 0.48
Hinduism	RC				RC			
**Marital status**								
Unmarried	−0.29	−0.04	0.508	−1.16, 0.57	−.003	−.001	0.990	−0.53, 0.52
Married	RC				RC			
**Institution type**								
Public	−0.4	−0.04	0.497	−1.56, 0.76	−2.16	−0.31	0.000	−2.87, −1.46
Private	RC				RC			
**Education**								
No formal education	−2.46	−0.18	0.001	−3.94, −0.97	0.6	0.06	0.201	−0.32, 1.51
Primary	−2.06	−0.36	0.000	−2.94, −1.17	−0.19	−0.04	0.509	−0.74, 0.37
Secondary	−1.28	−0.23	0.001	−2.06, −0.51	−0.01	−.002	0.978	−0.48, 0.47
Higher secondary or above	RC				RC			
**Employment type**								
Part-time	0.62	0.03	0.539	−1.36, 2.59	1.07	0.07	0.080	−0.13, 2.27
Full time	0.25	0.05	0.494	−0.47, 0.98	0.38	0.09	0.090	−0.06, 0.82
Temporal	RC				RC			
**Employment experience (Years)**								
Below 5	0.55	0.09	0.268	−0.42, 1.52	0.08	0.02	0.797	−0.51, 0.67
5 to 10	0.51	0.09	0.191	−0.26, 1.28	0.14	0.03	0.559	−0.33, 0.61
Above 10	RC				RC			
**Working hours per day**								
Up to 10	RC				RC			
Above 10	0.85	0.14	0.008	0.23, 1.46	0.46	0.1	0.017	0.08, 0.84
**Idea about the HACCP system**								
No	RC				RC			
Yes	1.54	0.16	0.003	0.54, 2.53	0.94	0.13	0.003	0.33, 1.55
**Idea about food safety authority**								
No	RC				RC			
Yes	0.25	0.04	0.403	−0.33, 0.82	0.49	0.11	0.006	0.14, 0.84
**Food safety training**								
No	RC				RC			
Yes	−0.52	−0.06	0.291	−1.5, 0.45	0.65	0.09	0.032	0.06, 1.24
**Health perception**								
Poor	RC				RC			
Average	0.58	0.11	0.092	−0.09, 1.25	0.15	0.04	0.483	−0.26, 0.56
Good	0.67	0.11	0.146	−0.24, 1.58	0.82	0.17	0.004	0.26, 1.37
**Knowledge Score**	NI				0.13	0.17	0.000	0.07, 0.19

Note: † = Adjusted R square: 0.16; ‡ = Adjusted R square: 0.45: NI= Not indicated; CI= Confidence intervals; RC= Reference class.

## 4 Discussion

This study aimed to evaluate the level of food safety awareness and adherence to food safety practices among institutional food handlers in Bangladesh. The results indicated that the participants possessed a moderate level of knowledge (61%) and adherence (64%) to food safety practices. There has been only one previous study conducted on the food safety knowledge and practice of hospital food service workers in Bangladesh. This study reported that workers had moderate knowledge (59.7%) and practice (50%), which is slightly lower than the findings of the current study [22]. The findings are in line with earlier research in Pakistan (60.0% knowledge and 58.0% practice) [7], Malaysia (61.7% knowledge and 53.2% practice) [37], Lebanon (59.2% knowledge and 83.2% practice) (although practice score was higher) [9], and lower than Sudan (70.1% knowledge and 74.4% practice) [38]. The discrepancies reported among studies may be ascribed to disparities in study design, study sites, cutoff thresholds, training, and the accessibility of information pertaining to food hygiene and safety. The findings of this study underscore the continued significance of prioritizing and improving the understanding and implementation of food safety among institutional food handlers in Bangladesh.

According to this study, food handlers practiced appropriate personal hygiene and sanitation, as would be expected of food handlers who should have received ongoing hand hygiene training. Similar findings have been discovered in prior studies [9,39]. However, the findings differed from those of a Pakistan-based study [7]. For example, 25% of 300 food handlers at the University of Punjab's various canteens still do not use soap when washing their hands, and 55.9% do not wear proper uniforms while on the job. Hand washing properly and thoroughly is crucial in reducing the spread of harmful bacteria from workers to food [40]. Hand hygiene is more crucial for preventing illness than cleaning and disinfecting surfaces that come into contact with food [41]. However, the present study revealed limited knowledge among institutional food handlers in Bangladesh regarding the foodborne pathogens associated with specific foodborne illnesses. This finding is consistent with a recent investigation carried out in Bangladesh, encompassing a sample of 191 individuals employed in the food service sector across seven hospitals located in Dhaka and Chattogram. In that study, a majority of participants inaccurately identified foodborne pathogens and their modes of transmission [22]. Our outcomes are also in line with those of earlier research carried out in Ghana [3], and Brazil [42], all of which found a lack of knowledge about foodborne pathogens. It's possible that a lack of scientific terminology in the training materials and educational background contributed to respondents' inability to correctly identify and link food with the transmission of certain foodborne pathogens. Hence, it is recommended that the implementation of comprehensive training initiatives targeting food handlers, encompassing the elucidation of the etiology of foodborne diseases, the modes of transmission of these diseases, and the optimal strategies for their prevention and management, may effectively contribute to the mitigation of foodborne outbreaks.

The present study revealed notable disparities in the mean food safety knowledge scores among various sociodemographic variables, aligning with other research findings [9,22]. Food handlers in rural areas, in particular, were found to have significantly more knowledge than their urban counterparts. In contrast, Islam et al. (2023) and Hamed et al. (2020) found that food handlers in urban areas were more knowledgeable about food safety than those in rural areas [17,43]. The observed discrepancies can be attributed to variations in study populations, methodologies, or regional differences, which emphasize the intricate nature of the association between sociodemographic variables and food safety knowledge. The potential explanations of our findings could be that food handlers in rural areas may have more experience working with food because they are more likely to be employed in agriculture or to be actively involved in the preparation of food for their families and communities. This experience could translate into a greater understanding of food safety knowledge.

Our research findings indicate that individuals who were familiar with the Hazard Analysis and Critical Control Points (HACCP) system exhibited a greater understanding of food safety principles and were more likely to follow safe food handling practices when compared to those who were not aware of HACCP. This result aligns with a study conducted by Osaili and colleagues in 2017, which demonstrated that individuals who recognized the importance of HACCP in ensuring food safety achieved higher scores in terms of their food safety knowledge [39]. In contrast to our findings, a study conducted by Al Banna and their team in 2022 reported that perceptions of the HACCP system did not significantly correlate with food safety knowledge and practices according to their multiple linear regression models [22]. The difference in our results compared to the findings of Al Banna et al. (2022) emphasizes the importance of further research to investigate the complex variables that impact the relationship between HACCP knowledge and food safety practices. However, it is worth noting that the implementation of HACCP has been slow in Bangladesh due to the high cost associated with its nationwide adoption [44]. This financial barrier may impact the widespread adoption of HACCP and contribute to variations in findings across different regions. Nonetheless, despite these challenges, our study provides support for the recommendation that institutional food handlers should consider adopting and implementing the HACCP strategy. This approach can provide food-service staff with the necessary knowledge and ongoing training in food hygiene practices, improve proper hygienic techniques, and engage each individual in maintaining food safety standards [45,46].

Additionally, our investigation uncovered that individuals holding at least a higher secondary education level displayed a notably greater proficiency in food safety knowledge in contrast to those with no formal education or only primary or secondary education. These findings parallel those of Alemayehu et al. (2021) and Moreb et al. (2017), both of which established that food handlers with just a primary education were less likely to possess a sufficient understanding of food safety compared to those with at least a secondary education [47,48]. The variation in knowledge levels can be ascribed to the fact that educated food handlers typically enjoy more extensive access to information about food safety, giving them a more comprehensive grasp of the subject compared to their less-educated counterparts. Having a higher level of education allows food handlers to access additional written materials on food safety, such as pamphlets, posters, and leaflets, which likely contribute to their enhanced knowledge [47]. Furthermore, our findings highlight the significant aspect of rapid knowledge and skill acquisition through training programs. Research has shown that food handlers who possess higher levels of education have a greater ability to absorb and comprehend information during training sessions [49].

In line with prior research [[50], [51], [52]], our results affirm the connection between undergoing food safety training and the adoption of proper food safety practices. The consistent validation of this relationship through several research emphasizes the crucial significance of formal training in influencing the practices of food handlers. However, it is crucial to acknowledge that a mere 9.6% of the food handlers in our study had received food safety training. This data highlights a significant gap in the present strategy for assuring the proficiency of institutional food handlers. This underscores the pressing need for a broader implementation of food safety training programs for institutional food handlers. Such a step is essential for effectively addressing food safety concerns and reducing the risks associated with foodborne illnesses.

Similar to findings from other studies conducted in Bangladesh [22] and Iran [53], our study revealed that participants working in private institutions exhibited higher scores in food safety practices compared to those in government institutions. Low- and middle-income countries, including Bangladesh, have insufficient institutional capability, poor governance, limited equipment availability, and a lack of skilled staff [54]. Therefore, the public sector must be included and consulted to guarantee inclusive and motivated efforts to tackle such challenges [55]. However, Osaili et al. found that private hospitals, unlike public hospitals, send staff to seminars and training sessions to learn about food safety [39]. Due to market competition, private institutions are typically more efficient and responsive to consumer requirements, and because their consumers have high expectations for consuming safe foods, they emphasize safe food handling practices [39]. These observations highlight the efficiency and adaptability of private institutions, driven by market competition, and suggest potential improvements in public institutions. More financing, focused training, compliance incentives, strong regulatory leadership, and better management structures—all of which bridge the gap between the public and private sectors—are needed to improve food safety standards in public institutions.

Our present research did have certain limitations, so first of all, this study was limited to a small number of institutions and it cannot be generalized to all institutional food handlers in Bangladesh. Further research, especially with food service workers from all around Bangladesh, is strongly suggested. No causal presumptions about the results could be drawn because the cross-sectional survey data employed in the current study prevented it. Non-probability sampling was used to choose the institutions and the participants, which might bias the results. The sample from private institutions is substantially smaller than the sample from public institutions. More research with diverse samples is needed to provide more strong statistical comparisons between food service workers from public and private institutions. Information provided by respondents was utilized to assess food safety practices rather than observational checklists, which might bring bias into the reporting process. To obtain a more precise assessment of food handlers' practices, it would be essential to conduct observations throughout their entire workday. Furthermore, it is important to acknowledge that the presence of a researcher in the work environment may influence participants' questionnaire responses.

## 5 Conclusion

This study found moderate knowledge and practices of food safety among institutional food handlers. These findings highlight the need to provide institutional food handlers with comprehensive training from institutions and government organizations that are necessary to increase their understanding of food safety and, in turn, improve their handling practices. Food handlers can be obligated to attend training sessions, which may be administered via legislative control of food safety rules by local governments as well as the BFSA. The results of this study also highlighted the importance of assessing the impact of the current national safe food handling course in light of the diverse cultural backgrounds and educational attainment levels of the people who work in the food service industry. To ensure that higher safety standards are adhered to in these facilities, institutions can consider recruiting personnel with advanced degrees. Managers, supervisors, and operators in all institutions must assume key responsibilities in implementing the HACCP system. They should possess a strong understanding of HACCP plans, principles, and relevant standard operating procedures. Finally, the government can establish and enforce laws that require both public and private institutions; the government can provide resources such as a planned funding mechanism for food safety equipment, infrastructure upgrades, transparency, and more meaningful directed actions to meet high food safety standards.

## Ethical consideration

The present study adhered to the ethical principles specified in the Helsinki Declaration of 2013 concerning the inclusion of human participants. Prior to its initiation, the research completed a thorough review process and received ethical clearance from the Institutional Ethical Committee of Patuakhali Science and Technology University, Bangladesh, with the approval number PSTU/IEC/2022/32 (3). This approval indicates that the study was carried out following ethical norms and principles, thereby taking care of the well-being and rights of the participants.

## Funding

The present study did not receive any explicit financial support from public, commercial, or not-for-profit funding bodies.

## Data availability

The data that support the findings of this study are available from the corresponding author upon reasonable request.

## CRediT authorship contribution statement

**Aysha Siddiky:** Writing – review & editing, Writing – original draft, Visualization, Methodology, Investigation, Data curation, Conceptualization. **Kakali Mollick:** Writing – review & editing, Visualization, Methodology, Data curation. **Md. Aktarujjaman:** Writing – review & editing, Methodology, Data curation. **Farhadul Islam:** Writing – review & editing, Visualization. **Mohammed A. Mamun:** Writing – review & editing, Visualization. **Nitai Roy:** Writing – review & editing, Writing – original draft, Visualization, Validation, Supervision, Software, Resources, Project administration, Methodology, Investigation, Formal analysis, Conceptualization.

## Declaration of competing interest

The authors declare that they have no known competing financial interests or personal relationships that could have appeared to influence the work reported in this paper.
