# Supplementary material for: Determinants of food safety knowledge and practices among food handlers in Bangladesh: An institution-based cross-sectional study
Source: Heliyon. 2024 Feb 10;10(4):e25970. doi: 10.1016/j.heliyon.2024.e25970 (PMC10878943; doi:10.1016/j.heliyon.2024.e25970)
Supplement: Multimedia component 1 [file mmc1.pdf]

সংগৃহীত তথ্য/ ডেটা কঠোরভাবে গোপন রাখা হবে এবং ফলাফলগুলি শুধুমাত্র গবেষণার উদ্দেশ্যে ব্যবহার করা হবে
আপনি কি এই জরিপে অংশগ্রহণ করতে ইচ্ছুক?
ক) হ্যাঁ খ) না
স্বাক্ষর.................................

ক-অংশ: খাদ্য হ্যান্ডলার সম্পর্কে সাধারণ তথ্য

১.আপনার লিঙ্গ কি?	ক) পুরুষ খ) মহিলা
২.আপনার বয়স কত?	……………………. বছর
৩.আপনার বাসস্থান কোথায়?	ক) শহর খ) গ্রাম
৪. আপনি কোন ধর্ম অনুসারি?	ক) ইসলাম খ) হিন্দুধর্ম
৫.আপনার বৈবাহিক অবস্থা কেমন?	ক) অবিবাহিত খ) বিবাহিত
৬. আপনার মাসিক আয় কত?	…………………..টাকা
৭. আপনার শিক্ষাগত যোগ্যতা কি?	ক) কোন প্রাতিষ্ঠানিক শিক্ষা নাই খ) প্রাথমিক<br>গ) মাধ্যমিক ঘ) উচ্চ মাধ্যমিক বা তার উপরে
৮. আপনি কোন ধরনের প্রতিষ্ঠানে কাজ করেন?	ক) সরকারী খ) বেসরকারী
৯. আপনার চাকরির অভিজ্ঞতা কত বছরের?	……………………………..বছর
১০. আপনার কর্মসংস্থানের ধরন কোনটি?	ক) খণ্ডকালীন খ) স্থায়ী গ) অস্থায়ী
১১. আপনি প্রতিদিন কত ঘন্টা কাজ করেন?	………………..ঘন্টা
১২. আপনার HACCP সিস্টেম সম্পর্কে ধারণা আছে?	ক) হ্যাঁ খ) না
১৩. খাদ্য নিরাপত্তা কর্তৃপক্ষ সম্পর্কে আপনার কোন ধারণা আছে?	ক) হ্যাঁ খ) না
১৪. খাদ্য নিরাপত্তা বিষয়ে আপনার কোন প্রশিক্ষণ আছে?	ক) হ্যাঁ খ) না
১৫. আপনার স্বাস্থ্যের অবস্থা কেমন?	ক) মন্দ খ) মোটামুটি গ) ভাল

খ- অংশ: কর্মচারীদের কাজের সন্তুষ্টি (কোনটি প্রযোজ্য তা ডানদিকের যে কোন একটি ঘরে (✓) চিহ্ন দিয়ে নির্দেশ করুন)

প্রশ্ন	হ্যাঁ	না	জানি না
১. যদি একটি পেশা নির্বাচন করার সুযোগ দেওয়া হয়, আপনি কি একই পেশা গ্রহণ করতে চান?			
২ যদি আরও সুবিধাজনক কর্মসংস্থানের সুযোগ উপস্থাপন করা হয়, আপনি কি তা গ্রহণ করতে আগ্রহী হবেন?			
৩. কর্মক্ষেত্রে কি খাদ্য নিরাপত্তার মান বজায় রাখার জন্য প্রয়োজনীয় সমস্ত ব্যাবস্থা রয়েছে?			
৪. প্রতিষ্ঠানের অন্যান্য কর্মচারীরা কি রান্নাঘরের কর্মীদের প্রতি শ্রদ্ধাশীল?			
৫. ভোক্তাদের পরিবেশিত খাবারগুলি কি তাদের স্বাস্থ্যের জন্য বিপজ্জনক?			

গ -অংশ: বাংলাদেশে প্রাতিষ্ঠানিক খাদ্য হ্যান্ডলারদের খাদ্য নিরাপত্তা সম্পর্কিত জ্ঞান (কোনটি প্রযোজ্য তা ডানদিকের যে কোন একটি ঘরে (✓) চিহ্ন দিয়ে নির্দেশ করুন)

প্রশ্ন	সত্য	মিথ্যা	জানি না
১. টাকা সরবরাহ করার পরে আপনার হাত ধোয়া অত্যন্ত গুরুত্বপূর্ণ।			
২. টেবিল পরিষ্কার করার পরে আপনার হাত ধোয়া অত্যন্ত গুরুত্বপূর্ণ।			
৩. হাঁচি দেওয়ার পর হাত পরিষ্কার করা জরুরি।			
৪. টয়লেট ব্যবহারের পরে আপনার হাত পরিষ্কার করা অপরিহার্য।			
৫. আপনার হাত ধোয়ার জন্য সময় প্রয়োজন প্রায় 20 সেকেন্ড।			
৬.খাবার তৈরি করার আগে, আপনার হাত ধোয়া অত্যন্ত গুরুত্বপূর্ণ।			
৭. কাঁচা মাংস নিয়ে কাজ করার পরে আপনার হাত ধোয়া অত্যন্ত গুরুত্বপূর্ণ।			
৮. কাঁচা খাবার পরিবেশনের আগে এবং পরে হাত ধোয়া খাদ্য দূষণের ঝুঁকি কমায়।			
৯. খাবার পরিবেশন করার সময় গ্লাভস পরা খাদ্য দূষণের ঝুঁকি কমায়।			

১০. ডিটারজেন্ট দিয়ে থালাবাটি পরিষ্কার করলে দূষণের ঝুঁকি বাড়ায়।			
১১. কাঁচা খাবার নিয়ে কাজ করার সময় খাওয়া এবং পান করা খাদ্য দূষণের ঝুঁকি বাড়ায়।			
১২. ক্রস-দূষণ কমাতে কাঁচা এবং রান্না করা খাবার আলাদাভাবে রাখা উচিত।			
১৩. দূষিত খাবার দ্বারা টাইফয়েড জ্বর ছড়াতে পারে।			
১৪. দূষিত খাবারের মাধ্যমে এইডস ছড়াতে পারে।			
১৫. দূষিত খাবারের মাধ্যমে রক্তাক্ত ডায়রিয়া ছড়াতে পারে।			
১৬. *Salmonella* বিভিন্ন খাদ্যবাহিত রোগজীবাণুগুলির মধ্যে একটি হিসাবে বিবেচিত হয়।			
১৭. *E. coli* বিভিন্ন খাদ্যবাহিত রোগজীবাণুগুলির মধ্যে একটি হিসাবে বিবেচিত হয়।			
১৮. *Shigella* বিভিন্ন খাদ্যবাহিত রোগজীবাণুগুলির মধ্যে একটি হিসাবে বিবেচিত হয়।			
১৯. *Bacillus cereus* বিভিন্ন খাদ্যবাহিত রোগজীবাণুগুলির মধ্যে একটি হিসাবে বিবেচিত হয়।			
২০. *Hepatitis A* বিভিন্ন খাদ্যবাহিত রোগজীবাণুগুলির মধ্যে একটি হিসাবে বিবেচিত হয়।			
২১.সবচেয়ে স্বাস্থ্যবান খাদ্য সরবরাহকারী ত্বক, নাক এবং মুখেও জীবাণু পাওয়া যায়।			
২২. পচনশীল খাদ্য সংরক্ষণের জন্য আদর্শ তাপমাত্রা হল ৫ ডিগ্রি সেলসিয়াস।			
২৩. যে খাবারগুলো গরম এবং খাওয়ার জন্য প্রস্তুত সেগুলোর সংরক্ষণ তাপমাত্রা হল 65 ডিগ্রি সেলসিয়াস।			
২৪. হিমায়িত প্রক্রিয়া সমস্ত ব্যাকটেরিয়াকে কার্যকরভাবে নিষ্ক্রিয় করে যা খাদ্যজনিত অসুস্থতার কারণ হতে পারে।			
২৫. কাঁচা মাংস ফ্রিজের নিচের শেলফে সংরক্ষণ করতে হবে।			
২৬. কাঁচা এবং রান্না করা খাবার একসাথে সংরক্ষণ করলে বিষক্রিয়া হয়।			

ঘ- অংশ: বাংলাদেশে প্রাতিষ্ঠানিক খাদ্য হ্যান্ডলারদের মধ্যে খাদ্য নিরাপত্তা বিষয়ক অভ্যাস (কোনটি প্রযোজ্য তা ডানদিকের যে কোন একটি ঘরে (✓) চিহ্ন দিয়ে নির্দেশ করুন)

**প্রশ্ন**	**হ্যাঁ**	**না**
১. প্যাকেটবিহীন, কাঁচা খাবার নিয়ে কাজ করার আগে এবং পরে আপনি কি সবসময় আপনার হাত ধুয়ে নেন?		
২. প্যাকেটবিহীন প্রস্তুত খাবার নিয়ে কাজ করার আগে এবং পরে উভয় ক্ষেত্রেই আপনি কি আপনার হাত ধুয়ে নেন?		
৩. খাবার তৈরি করার সময় আপনি কি গ্লাভস পরেন?		
৪. আপনি যখন প্যাকেটে মোড়ানো না এমন খাবার স্পর্শ করেন বা বিতরণ করেন তখন কি এপ্রোন ব্যবহার করেন?		
৫. আপনি যখন প্যাকেটে মোড়ানো না এমন খাবার স্পর্শ করেন বা বিতরণ করেন তখন কি মাস্ক ব্যবহার করেন?		
৬. প্যাকেটে মোড়ানো না এমন খাবার স্পর্শ করা বা বিতরণ করার সময় আপনি কি আপনার চুল ঢেকে নেন?		
৭. আপনি কি আঙুলের নখ বড় রাখেন?		
৮. আপনি কি সবজি কাটার আগে ধুয়ে নেন?		
৯. আপনি কি তাজা সবজি, ফল, এবং কাঁচা মাংসের জন্য আলাদা কাটিং বোর্ড ব্যবহার করেন?		
১০. আপনি কি কাঁচা মাংস বা মুরগির মাংস কাটার পরে ছুরিটি পরিষ্কার এবং জীবাণুমুক্ত করেন?		
১১. আপনি যখন সংক্রামক রোগে (জ্বর,সর্দি ডায়রিয়া, কাশি ইত্যাদি) আক্রান্ত হন তখন কি কাজ করেন?		
১২. আপনার হাতে ক্ষত বা কাটা থাকলে, আপনি কি কাজ চালিয়ে যান?		
১৩. আপনি কি স্বাভাবিক তাপমাত্রায় খাবার গলাতে দেন?		
১৪. আপনি কি প্রস্তুত খাবার স্বাভাবিক তাপমাত্রায় চার ঘণ্টার বেশি সময় রাখেন?		
১৫. আপনি কি খাবার তৈরির আগে এবং পরে খাবার তৈরির জায়গা ধুয়ে ফেলেন?		
