# Supplementary material for: Determinants of food safety knowledge and practices among food handlers in Bangladesh: An institution-based cross-sectional study
Source: Heliyon. 2024 Feb 10;10(4):e25970. doi: 10.1016/j.heliyon.2024.e25970 (PMC10878943; doi:10.1016/j.heliyon.2024.e25970)
Supplement: Multimedia component 2 [file mmc2.docx]

The information/data collected will be kept strictly confidential and the results will be used for research purposes only.

Are you willing to participate in this survey? A. Yes B. No

Signature:

**Section A. Socio-demographic Information of food handlers**

| 1. What is your gender? | A. Female B. Male |
| --- | --- |
| 2. How old are you? | ……………..... years |
| 3. Where is your residence? | A. Urban B. Rural |
| 4. What is your religion? | A. Islam B. Hinduism |
| 5. What is your marital status? | A. Unmarried B. Married |
| 6. What is your monthly income? | ……………… BDT |
| 7. What level of education have you achieved? | A. No formal education B. Primary C. Secondary  D. Higher secondary or above |
| 8. What is the type of your institution? | A. Public B. Private |
| 9. How long is your employment experience? | ………………..years |
| 10. What is your employment type? | A. Part-time B. Full-time C. Temporal |
| 11. How many hours do you work per day? | …………………….hours |
| 12. Do you have any idea about the HACCP system? | A. Yes B. No |
| 13. Do you have any idea about food safety authority? | A. Yes B. No |
| 14. Do you have any food safety training? | A. Yes B. No |
| 15. How do you perceive your health? | A. Poor B. Fair C. Good |

**Section B. Employees’ work satisfaction (Indicate with a checkmark (✓) in the box to the right)**

| Questions | Yes | No | Don’t know |
| --- | --- | --- | --- |
| 1. If allowed to select a profession, would you want to pursue the same profession? |  |  |  |
| 2. If presented with a more advantageous employment opportunity, would you be inclined to accept it? |  |  |  |
| 3. Does the workplace possess all of the necessary conditions to ensure the upkeep of food safety standards? |  |  |  |
| 4. Are the other employees of the institution respectful of the kitchen staff? |  |  |  |
| 5. Are the foods served to individuals hazardous to their health? |  |  |  |

**Section C. Food safety knowledge of institutional food handlers in Bangladesh (Indicate with a checkmark (✓) in the box to the right)**

| Questions | True | False | Don’t know |
| --- | --- | --- | --- |
| 1. It is critical to wash your hands after dealing with currency. |  |  |  |
| 2. It is essential to cleanse your hands upon wiping down a table. |  |  |  |
| 3. It is imperative to cleanse your hands after sneezing. |  |  |  |
| 4. It is essential to cleanse your hands after using the toilet. |  |  |  |
| 5. The time required to wash your hands is about 20 seconds. |  |  |  |
| 6. Prior to making a meal, washing your hands is crucial. |  |  |  |
| 7. It's crucial to wash your hands following the handling of raw meat. |  |  |  |
| 8. Handwashing minimizes the chance of food contamination both before and after handling raw food. |  |  |  |
| 9. Wearing gloves while handling food helps to reduce the chance of food contamination. |  |  |  |
| 10. Utensils that have been cleaned with detergent increase the risk of contamination. |  |  |  |
| 11. Eating and drinking during raw food handling increases the risk of food contamination. |  |  |  |
| 12. Raw and cooked foods should be kept separate to minimize cross-contamination. |  |  |  |
| 13. Typhoid fever can be transmitted by contaminated food. |  |  |  |
| 14. AIDS is transmissible through contaminated food. |  |  |  |
| 15. Bloody diarrhea is transmissible through contaminated food. |  |  |  |
| 16. *Salmonella* is regarded as one of the various food-borne pathogens. |  |  |  |
| 17. *E. coli* is regarded as one of the various food-borne pathogens. |  |  |  |
| 18. *Shigella* is regarded as one of the various foodborne pathogens. |  |  |  |
| 19. *Bacillus cereus* is regarded as one of the various foodborne pathogens. |  |  |  |
| 20. *Hepatitis A* is regarded as one of the various foodborne pathogens. |  |  |  |
| 21. Microbes can be found on the skin, nose, and mouth of even the healthiest food handlers. |  |  |  |
| 22. The ideal temperature for storing perishable food is 5 ° C. |  |  |  |
| 23. The storage temperature for hot, ready-to-eat food is 65 °C. |  |  |  |
| 24. The freezing process effectively inactivates all bacteria that have the potential to cause foodborne illnesses. |  |  |  |
| 25. Raw meat should be stored on the bottom shelf of the refrigerator. |  |  |  |
| 26. Storing raw and cooked food together causes poisoning. |  |  |  |

**Section D. Food safety practices among institutional food handlers in Bangladesh (Indicate with a checkmark (✓) in the box to the right)**

| Questions | Yes | No |
| --- | --- | --- |
| 1. Do you always wash your hands before and after working with unpackaged, raw foods? |  |  |
| 2. Do you always wash your hands both prior to and subsequent to handling unpackaged prepared food? |  |  |
| 3. Do you wear gloves while preparing food? |  |  |
| 4. When touching or passing out unwrapped foods, do you wear an apron? |  |  |
| 5. When touching or passing out unwrapped food, do you use a mask? |  |  |
| 6. Do you cover your hair when touching or distributing unwrapped food? |  |  |
| 7. Do you let your fingernails grow? |  |  |
| 8. Do you rinse vegetables prior to slicing them? |  |  |
| 9. Do you utilize separate cutting boards for fresh vegetables, fruits, and raw meat? |  |  |
| 10. Do you clean and disinfect the knife after slicing raw meat or chicken? |  |  |
| 11. Do you work when you are suffering from infectious diseases (flu, cold, diarrhea, coughing, etc.)? |  |  |
| 12. If you have lesions or cuts on your hands, do you continue work? |  |  |
| 13. Do you allow food to thaw at room temperature? |  |  |
| 14. Do you leave prepared food out at room temperature for longer than four hours? |  |  |
| 15. Do you wash food contact surfaces prior to and following food preparation? |  |  |
